# Olmesartan-Induced Enteropathy: A Case Report

**DOI:** 10.7759/cureus.40342

**Published:** 2023-06-12

**Authors:** Dharmikkumar Jadvani, Pinky Jha, Meenu Singh

**Affiliations:** 1 Internal Medicine, Byramjee Jeejeebhoy (BJ) Medical College, Ahmedabad, IND; 2 Internal Medicine, Medical College of Wisconsin, Milwaukee, USA; 3 General Internal Medicine, University of Utah, Salt Lake City, USA

**Keywords:** diarrhea and abdominal pain, adverse drug effect, celiac sprue, villous atrophy, olmesartan-induced enteropathy

## Abstract

Patients with olmesartan-induced enteropathy, a rare illness, frequently endure prolonged diarrhea and weight loss with no apparent cause. Because this adverse event's clinical and histological characteristics mimic those of other small intestine illnesses, it can be challenging to recognize it in a timely manner. We report a case of olmesartan-induced enteropathy in a 58-year-old male who had been on olmesartan for several years. Recently, during his travel to Greece, he developed diarrhea lasting several weeks. This was accompanied by a significant weight loss of 35 lbs, acute kidney injury, and hypokalemia. Extensive negative workup, including esophagogastroduodenoscopy (EGD) with normal biopsy of esophagus, stomach, duodenum, and terminal ileum, and colonoscopy with biopsies, autoimmune serologies, and infectious disease workup, led to a diagnosis of olmesartan-induced enteropathy as a diagnosis of exclusion. Diarrhea improved/resolved within a few days after stopping olmesartan in our patient.

## Introduction

Olmesartan medoxomil, an angiotensin II receptor blocker (ARB), is the prodrug of olmesartan. It was licensed to treat hypertension in the USA in 2002 and the European Union in 2003 [1]. Olmesartan is a potent, commonly prescribed first-line antihypertensive medication in adults and the pediatric population > six years of age. About 1,163,607 people received an estimated 4,458,453 prescriptions for olmesartan in the year 2020 in the USA [2].

Olmesartan has relatively few side effects. Olmesartan-induced enteropathy was first reported in 2012 in a case series of 22 patients. Since then, more cases associated with olmesartan and with other ARBs (irbesartan, valsartan, telmisartan) have been reported [3].

Most cases have been associated with celiac sprue-like villous atrophy on intestinal biopsy. The precise mechanism of pathogenesis has yet to be determined. A possible mechanism is postulated to inhibit the intestinal immune suppressive effect of transforming growth factor-beta (TGF-β), with a consequent increase of intestinal T-cell inflammation [3] leading to cellular damage and malabsorption.

The lack of awareness regarding this disease entity leads to underdiagnosis and overutilization of healthcare resources with unnecessary patient suffering, including hospital admissions for a condition that usually improves with discontinuation of the offending drug, as was seen in the case discussed below.

## Case presentation

This is a case of a 58-year-old male with a past medical history of hypertension, gastroesophageal reflux disease, hypothyroidism, and cholecystectomy who presented to the hospital with complaints of progressive diarrhea, especially after any oral intake leading to fear of eating. The patient was vacationing in Greece three months previously when the diarrhea started. He mentioned drinking tap water but denied any sick contacts or changes in diet or his medications. Diarrhea persisted after returning to America, and he went to his primary care provider, who empirically started the patient on metronidazole. Metronidazole helped improve the symptoms transiently. Given ongoing diarrhea, an esophagogastroduodenoscopy (EDG) and colonoscopy were performed one week prior to hospital admission. The results ruled out celiac sprue as biopsies of the esophagus, stomach, duodenum, and terminal ileum showed normal findings, but colonoscopy revealed a few non-bleeding diverticula and identified a single 3 mm sessile rectal polyp, which upon biopsy, exhibited epithelial hyperplasia. Worsening diarrhea, fear of eating, lightheadedness, and 35 lb weight loss led to hospitalization. The patient denied any fever, chills, vomiting, melena, abdominal pain, or blood in the stool. Home medications included olmesartan, levothyroxine, and metoprolol. On admission, the patient had bradycardia (60-70/min), dry mucous membranes, and hyperactive bowel sounds without abdominal tenderness. Initially, infectious causes were suspected, and he was managed accordingly with fluid resuscitation and treating existing conditions. An extensive workup was done to find the underlying etiology, which included a complete blood count and comprehensive metabolic panel, which was in the normal range except for hypokalemia and acute kidney injury with elevated Cr likely due to dehydration secondary to diarrhea. The hepatitis panel, bile acids, tissue transglutaminase antibody IgA, and 5 HIAA were all in the normal range. Infectious studies, including stool *C. difficile*, ova/parasite, salmonella, shigella, *campylobacter jejuni*, *E. coli*, *Vibrio*, *Aeromonas*, *Plesiomonas*, and *Giardia* antigen, were all negative.

Table 1 depicts pertinent lab values for the case report.

**Table 1:** Laboratory findings of the case report

Test Name	Result	Reference Value
Potassium (K)	2.9 mmol/L	3.5-5.5 mmol/L
Creatinine (Cr)	1.59 mg/dL	0.72-1.25 mg/dL
Thyroid-stimulating hormone (TSH)	86 mU/L	0.27-4.20 mU/L
Free T4	0.7 ng/dL	0.9-1.7 ng/dL
Serum lipase	54 U/L	8-78 U/L
Serum osmolality	286 mOsm/kg	280-303 mOsm/kg
C-reactive protein (CRP)	0.5 mg/dL	0.0-0.8 mg/dL
Vasoactive intestinal peptide (VIP)	<13 pg/mL	0-60 pg/mL
Calcitonin	<2 pg/mL	0.0-7.5 pg/mL
Gastrin	16 pg/mL	0-100 pg/mL
Pancreatic elastase	>800 ug/g	2-100 ug/g
Serum cortisol	12.4 mcg/dL	5-25 mcg/dL
Stool osmolarity	365 mOsm/kg	280-303 mOsm/kg
Lactoferrin	Positive	
Calprotectin	331 ug/g	10-50 μg/mg
24-hour fecal fat	2.6 g/d	0.0-6.0 g/d

CT of the abdomen pelvis revealed no abnormal findings concerning GI pathology, given that most of the workup was inconclusive conservative measures, including dietary modifications and withholding known offending agents like omeprazole and non-steroidal anti-inflammatory drugs, were done without any improvement. Finally, olmesartan was held, and the patient's symptoms improved significantly within two days. On follow-up one month later, his symptoms and gastrointestinal inflammatory markers, including fecal calprotectin, had normalized to 30 (normal <45).

## Discussion

**Figure 1:**
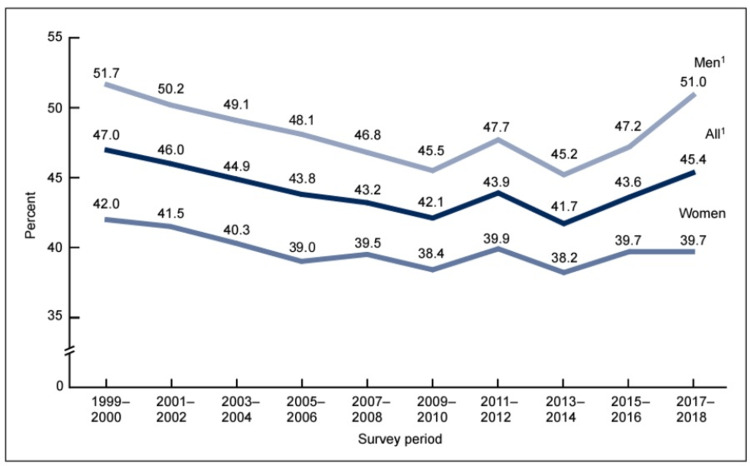
Age-adjusted trend in hypertension prevalence among adults aged 18 and over by sex: USA, 1999-2018 ^1^Significant quadratic trend from 1999 through 2018.

Diarrhea is a common side effect of medications. The prevalence of hypertension among adults aged 18 and older in the USA was 45.4% from 2017 to 2018 (Figure 1) [4], with an estimated global prevalence of 30% in adults.

Olmesartan is an ARB. According to a 2013 FDA report, olmesartan can cause a "sprue-like enteropathy" that manifests as severe persistent diarrhea and weight loss months to years after treatment begins, with the risk increasing after two years of use [5].

A similar timeline of experiencing the adverse drug reaction was seen in our patient, also approximately two years ago. In the literature, olmesartan-induced enteropathy has an estimated incidence of <0.05% of olmesartan users [3].

Olmesartan causes malabsorptive enteropathy; however, the precise processes by which this occurs are still not understood. The initial data point to cell-mediated immunity harm due to the delay in enteropathy after olmesartan usage. Two pathways have been suggested: (1) the inhibition of the transforming growth factor by ARBs, which increases the activity of T-cells, and (2) the disproportionate activation of angiotensin II receptor type 2 (AT2) receptors by angiotensin II after blocking AT1 receptors with olmesartan, which causes enterocyte apoptosis [6]. Laboratory evaluation predominantly reveals a severe malabsorption process which includes anemia, hypoalbuminemia, electrolyte imbalance, and vitamin deficiencies in cases of olmesartan-induced enteropathy. Given the overlapping clinical and histological patterns with celiac disease, negative celiac serology is imperative in diagnosing olmesartan-induced enteropathy [1].

In a French cohort of 4,546,680 patients, those taking olmesartan for one to two years (adjusted risk ratio 3.7, 95% CI 1.8-7.3) and those taking it for more than two years (adjusted risk ratio 10.6, 95% CI 5.0-22.5) experienced intestinal malabsorption severe enough to necessitate hospitalization significantly more frequently than those taking an ACE inhibitor [5]. The varying incidences among drugs within the same class may be related to the differences in affinity to AT1R versus AT2R and the amount of drug administered [7]. Treatment is simple and includes discontinuation of olmesartan and symptomatic diarrhea management while ruling out other possible causes.

Re-challenging patients with olmesartan is not recommended, though it has occurred unknowingly [1,8-10] with the resumption of symptoms exhibited. It is not unreasonable to consider replacing a patient's olmesartan with an alternative ARB if ARB is still desired as an antihypertensive of choice based on the patient's medical comorbid illnesses. Though if this isn't a priority, an agent from a different class may be the better option to avoid a class effect altogether.

Although the timeframe for diarrhea resolution varies, clinical improvement often happens a few days to a week after olmesartan is stopped [11-13], as was the case in our instance.

**Figure 2:**
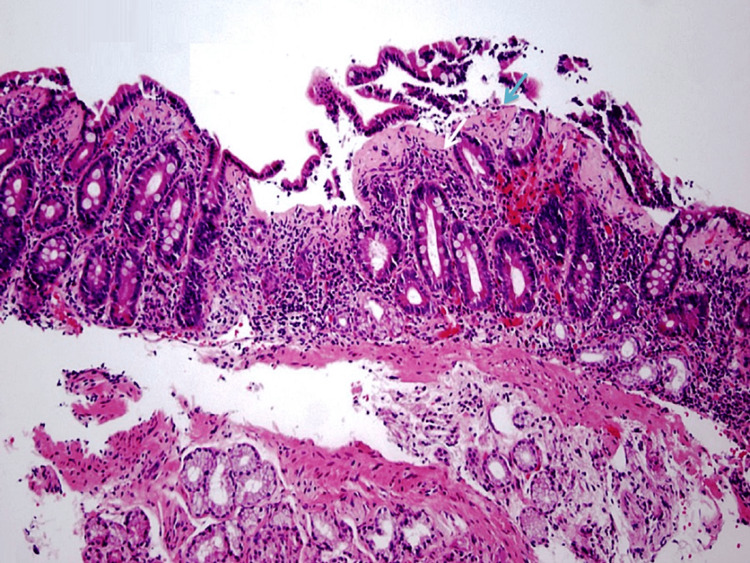
Pathologic examination of the duodenal biopsy specimen showed partial villous atrophy (blue arrow) with associated increased intraepithelial lymphocytes (white arrow). (Hematoxylin and eosin staining; 100x)

Minimal endoscopic changes were also noted in the colon and terminal ileum. Pathologic examination of the biopsy specimens from the duodenum showed chronic partial villous atrophy with increased intraepithelial lymphocytes (Figure 2) [14].

**Figure 3:**
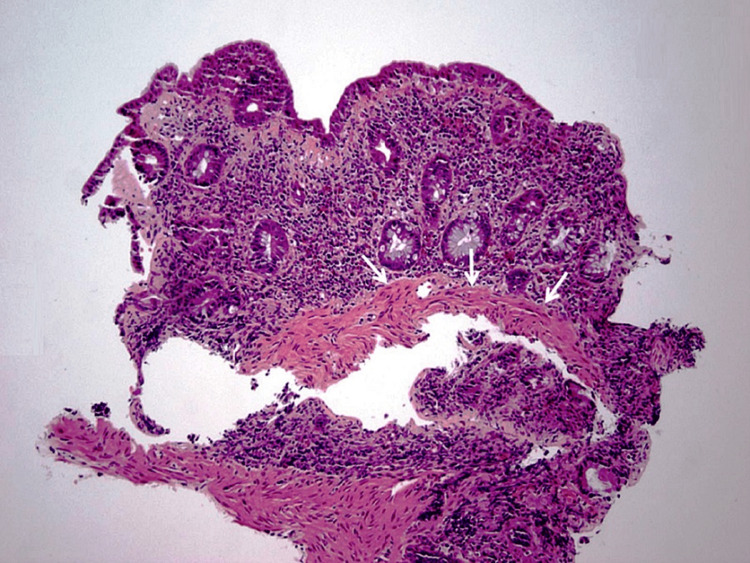
Duodenal biopsy showing a thickened subepithelial collagen band (arrows). (Hematoxylin and eosin staining; 100x)

A thickened subepithelial collagen band was another prominent histologic finding (Figure 3) [14].

The entities with overlapping histopathologic features are discussed below, and where possible, distinctions are noted (Table 2) [15].

**Table 2:** Possible histopathologic differences between ARB enteropathy and other entities ARB: angiotensin receptor blocker; IEL: intraepithelial lymphocytosis

Entity	Histopathologic features	Distinguishing features of ARB enteropathy
Celiac disease	Intraepithelial lymphocytosis, crypt hyperplasia, villous atrophy	IEL sometimes within, or close to, normal limits; collagen deposition frequent
Tropical sprue	Intraepithelial lymphocytosis, often worse in the terminal ileum than duodenum; often preserved architecture	Villi often flat; IEL sometimes within, or close to, normal limits; collagen deposition frequent
Autoimmune enteropathy	Variable features: villous atrophy, possible intraepithelial lymphocytosis, loss of goblet cells, loss of Paneth cells	No known histopathologic distinguishing features
Crohn disease	Patchy active inflammation, intraepithelial lymphocytosis, granulomas, variable architectural distortion	Granulomas not characteristic; diffuse involvement; collagen deposition frequent
Mycophenolate toxicity	Typically shows only increased crypt apoptosis; however, some cases may show intraepithelial lymphocytosis and/or villous atrophy	More diffuse and severe villous atrophy; more chronic and active inflammation; collagen deposition frequent

## Conclusions

Given that olmesartan is an extensively used medication for a widely prevalent medical condition, all physicians, especially primary care providers, hospitalists, and specialties, including gastroenterology and cardiology, should be aware of olmesartan-induced enteropathy. Overlooking the association of olmesartan causing a sprue-like enteropathy often leads to unnecessary extensive workups, interventions, continued patient suffering without the removal of the culprit medication, and associated poor outcomes. To further understand the diagnostic and therapeutic challenges connected with olmesartan-induced sprue-like enteropathy, prospective studies assessing the serological and histological profiles are required.
